# Dr. Otto Appley

**Published:** 1917-03

**Authors:** 


**Dr. Otto Appley,** Buffalo 1880, a practitioner of Damascus, Pa., died at the Deer Park Sanitarium, Port Jervis, N. Y., late in January, after an operation. He was 65 years old.

